# Neuroinflammatory mechanisms may help identify candidate biomarkers in chronic traumatic encephalopathy (CTE)

**DOI:** 10.17879/freeneuropathology-2025-6382

**Published:** 2025-07-14

**Authors:** Guneet S. Bindra, Shaheryar Asad, Jean Shanaa, Forshing Lui, Andrew E. Budson, Katherine W. Turk, Jonathan D. Cherry

**Affiliations:** 1 California Northstate University College of Medicine, Elk Grove, USA; 2 VA Boston Healthcare System, Boston, USA; 3 Boston University Alzheimer's Disease and CTE Centers, Boston University School of Medicine, Boston, USA; 4 Department of Neurology, Boston University School of Medicine, Boston, USA; 5 Department of Pathology and Laboratory Medicine, Boston University School of Medicine, Boston, USA; 6 Department of Anatomy and Neurobiology, Boston University School of Medicine, Boston, USA

**Keywords:** Chronic traumatic encephalopathy, Repetitive head impacts, Traumatic brain injury, Inflammatory signature, Biomarkers, Neuroinflammation

## Abstract

Chronic traumatic encephalopathy (CTE) is a neurodegenerative disease that can only be diagnosed post-mortem via pathological autopsy. The primary risk factor for CTE is a history of repetitive head impacts (RHI) received through contact sports including American football, hockey or soccer, military-related head injuries, or intimate partner violence. Recent findings have demonstrated that neuroinflammation is a critical compo-nent of early CTE pathogenesis and is likely part of the mechanism driving disease onset and progression. Additionally, the innate specificity, or ‘signature’, of a neuroinflammatory response may function as a dis-ease-specific marker for various neurodegenerative conditions. This would suggest an enormous repository of novel CTE biomarker candidates to be added to ongoing clinical trials, helping bolster diagnosis. However, few studies have truly leveraged immune mediators as candidate CTE markers. In this review, we argue and provide support that inflammatory mechanisms could serve as a viable source for novel biomarkers that are specific to CTE pathol-ogy. This includes an evaluation of inflammatory or damage-related markers such as CCL11 (C-C Motif Chem-okine Ligand 11, also known as Eotaxin-1), CCL21 (C-C Motif Chemokine Ligand 21) and GFAP (Glial Fibrillary Acidic Protein). We discuss the neuroinflammatory responses that give rise to these biomarkers in addition to the advantages and limitations of using each to diagnose CTE with particular attention to sensitivity and specifici-ty. Although further research is necessary to validate immune mediators, the latter show promise as diagnos-tic biomarkers for CTE and may also eventually serve as therapeutic targets for mitigating chronic inflamma-tion in at-risk populations.

## Abbreviations

**AD** - Alzheimer’s disease, **BBB** - Blood-brain barrier, **CCL2** - Chemokine ligand 2, **CCL11** - eotaxin-1, **CNS** - Central nervous system, **CRP** - C-reactive protein, **CSF** - Cerebrospinal fluid, **CTE** - Chronic traumatic encephalopathy, **DAMP** - Damage-associated molecular pattern, **DCE-MRI **- Dynamic contrast-enhanced magnetic resonance imaging, **DTI** - Diffusion tensor imaging, **ELISA** - Enzyme-linked immunosorbent assay, **ICAM1** - Intercellular adhesion molecule-1, **FDG** – Fluorodeoxyglucose, **FTD** - Frontotemporal dementia, **GFAP** - Glial fibrillary acidic protein, **HMGB1** - High mobility group box protein, **NBD** - Neurobehavioral dysregulation, **NfL** - Neurofilament light chain, **OPC** - Oligodendrocyte precursor cells, **P-tau **- Phosphorylated tau, **RHI** - repetitive head impacts, **TBI** - Traumatic brain injury, **TES** - Traumatic encephalopathy syndrome, **TNF-α **- Tumor necrosis factor-alpha, **TSPO** - Translocator protein, **TREM2** - Triggering receptor expressed on myeloid cells, **VCAM1** - Vascular cell adhesion molecule-1, **VEGF** - Vasoactive endothelial growth factor

## Introduction

Chronic traumatic encephalopathy (CTE) is a progressive neuropathological disease characterized by the perivascular accumulation of phosphorylated tau (p-tau) at the depths of cerebral sulci: (1) The largest risk factor for CTE is exposure to repetitive head impacts (RHI), whether concussive or non-concussive; (2) CTE pathology has been confirmed in patients with various RHI exposures including contact sports such as American football, hockey, rugby, boxing, and soccer (1,3,4); (3) Although sports-related head trauma has been recognized as the primary source of CTE cases in the literature, other types of exposure have been shown to be involved as well. There is emerging evidence of CTE cases in military veterans. Premier et al. demonstrated ten out of 225 (4.4 %) veteran brains had evidence of CTE (5). Although all ten of the veterans were found to also play a contact sports, other studies have observed CTE in veterans without a sports background (6); (4) There has also been concern for CTE in persons who experience long-term domestic violence. Dams-O’Connor et al. examined tissue from individuals exposed to intimate partner violence and did not observe CTE in their cohort (10); however, other case series and autopsy studies have identified CTE in individuals exposed to domestic abuse, suggesting that while less common, such exposures might still confer risk (11,12). Overall, while the incidence of CTE is likely much lower in exposures outside of contact sports, the risks are still not zero. However, a major limitation in our understanding of at-risk populations is that CTE can only be diagnosed postmortem. Although provisional in-life criteria have been suggested, coining the term traumatic encephalopathy syndrome (TES), they has not been fully validated and still remain research criteria (3). To date, there is no way to predict if RHI-exposed individuals will develop CTE years after exposure, since not all individuals with RHI exposure go on to acquire CTE (13,14). Therefore, there is great need for biomarkers to advance ante-mortem diagnosis of CTE.

The current focus around CTE biomarkers has primarily centered on structural protein markers, including tau proteins, neurofilament light protein (NfL) and glial fibrillary acidic protein (GFAP) (15). Despite some promising evidence, these proteins are often associated with other neurodegenerative conditions or acute injuries, hindering their specificity to CTE (19,20). Although certain phosphor epitopes of tau may be useful, tau markers may not generally be ideal for distinguishing between Alzheimer’s disease (AD) and CTE, as both are tauopathies (21). Currently, no adequate biomarkers exist to differentiate CTE from other forms of neurodegeneration. Interestingly, recent studies have suggested that the neuroimmune response might be distinct among related tauopathies (22). These findings, coupled with the advent of high-level genomic techniques like single cell RNA sequencing, have highlighted the enormous complexity and flexibility of the immune response to repetitive head trauma. It may therefore be useful to take advantage of this characteristic to identify novel sensitive and specific biomarkers that could effectively diagnose CTE in life. Herein we discuss neuroinflammation in CTE and examine specific inflammatory processes that may involve prospective novel biomarkers.

## Inflammation is strongly associated with CTE

Increasing evidence supports a role for inflammation as an early modifier or initiator of CTE disease progression, consistent with inflammation being a mechanistic driver of other neurodegenerative conditions. For instance, genomic studies have identified risk-stratifying immune-related loci in relation to AD, such as TREM2 (Triggering Receptor Expressed on Myeloid Cells 2) and CD33 (Cluster of Differentiation 33), (23,24), while TMEM106B (Triggering Receptor Expressed on Myeloid Cells 106B) and LRRK2 (Leucine-Rich Repeat Kinase 2) have been detected as immune-related variants affecting pathology in frontotemporal dementia (FTD) (25,26) and Parkinson’s disease (27,28), respectively. In fact, based on post-mortem genomic analysis of cerebellum tissue, Bieniek et al. reported that donors with RHI exposure and CTE diagnosis at autopsy tended to have a slight increase in the MAPT H1 haplotype, with less homozygous genotypes of the TMEM106B rs3173615 minor allele compared to non-CTE controls. Furthermore, immune signaling, such as through cytokine release from activated microglia, has been reported to promote tau hyperphosphorylation and to impair amyloid-β clearance (29).

Although the absence of a validated longitudinal or animal model limits the ability to experimentally demonstrate that CTE follows a similar pattern as other neurodegenerative states, post-mortem transcriptomic and computational analyses offer evidence that inflammation precedes pathology in CTE. In a large-scale mRNA-sequencing analysis of post-mortem CTE, Labadorf et al. reported upregulation of genes related to cytokine signaling, immune cell migration, and apoptosis in late stage CTE, while these same categories had inverse or reduced expression in early stage CTE. This suggests that early inflammatory responses could have a distinct mechanistic mechanistic function separate from the response to tauopathy. Notably, activation of immune pathways in early stages of CTE was positively correlated with duration of RHI exposure, suggesting inflammation as an initiating factor for CTE pathogenesis (32).

Similarly, Cherry et al. found that in post-mortem dorsolateral frontal cortex from donors with early stage CTE, immune-related genes were upregulated in sulci compared to neighboring gyri compared to RHI donors without CTE. These sulcal alterations in immune gene expression were also associated with RHI history and did not exclusively co-occur with pathology, further suggesting inflammation to occur upstream of tau deposition (33). Additional support comes from a recent study by Butler et al. who observed that in individuals under the age of 50 years with exposure to contact sports, cases that had exposure to RHI but no CTE pathology still had significant neuroinflammatory changes compared to control cases. Interestingly, the observed inflammatory changes were similar to those seen in cases with early stage CTE, suggesting that inflammation precedes the deposition of p-tau (34).

In addition, PET imaging of translocator protein (TSPO), which is expressed in the mitochondria of various neural cells (i.e., microglia, endothelial cells, astrocytes), showed increased TSPO in regions including right amygdala and bilateral supramarginal gyri for RHI-exposed football players (35,36). When examining TSPO via post-mortem immunoassay, Varlow et al. reported that despite the difference in TSPO density between CTE and controls not reaching statistical significance, there was a general trend of increased expression in CTE (37).

Taken together, these findings highlight that inflammation has a critical and variable role across CTE pathogenesis. Future studies that more precisely target these inflammatory processes and aim to better characterize the CTE immune signature may offer clearer insight into disease progression during life. This would consequently serve as a promising avenue for identifying CTE-specific biomarkers.

## Candidate neuroinflammatory markers for CTE

From the immune signature of CTE, candidate markers may emerge allowing distinction of CTE from other neurodegenerative conditions. Considering the preliminary evidence that inflammation precedes and contributes to CTE pathology, it could be worthwhile to evaluate proteins involved in the CTE immune profile for biomarker development. Here, we discuss current prospective neuroimmune biomarkers primarily derived from studies using postmortem fluids in neuropathologically confirmed CTE or in fluids from living individuals with a history of playing contact sports. As CTE can only be diagnosed after death, the postmortem studies are the only way to directly link a protein to confirmed CTE. However, as past work has demonstrated a strong correlation between more years of contact sports play and elevated risk of CTE (4), the prospective human studies offer additional insight into other targets that are likely to be involved in the CTE process, pending neuropathologic follow ups. Prospective markers are summarized in **Table 1**.

**Table 1:** Overview of human-based studies on prospective immune markers

Study	Immune protein(s)	Study design	Study populations and sample sizes	Significant findings
Cherry et al. 2017 (52)	CCL11	ELISA of postmortem dorsolateral frontal cortex (DLFC) and CSF analysis	23 CTE, 50 AD, and 18 non-athlete controls. All were neuropathologically confirmed.	CCL11 was elevated in DLFC of CTE compared to AD and controls. Same results seen upon CSF analysis. Receiver operator characteristics (ROC) curve analysis showed specificity of CCL11 to CTE. CCL11 correlated with RHI exposure duration.
Cherry et al. 2022 (22)	CCL21 CCL11 CXCL5 CXCL13 GMCSF CCL17	Multiplex ELISA of postmortem anterior cingulate grey matter and CSF analysis	40 CTE, 28 AD, 20 progressive supranuclear palsy, 20 corticobasal degeneration, 19 argyrophilic grain disease. All were neuropathologically confirmed.	From 71 immune proteins, CCL21 had the strongest correlation with CTE. CXCL5, CXCL13, GMCSF, and CCL17 had significant association with CTE based on ROC analysis. CSF analysis showed CCL21 to be more significantly increased in CTE samples than in AD samples.
Vig et al. 2023 (59)	CCL11	Immunoassay of postmortem vitreous humor	15 CTE, 7 AD, 10 with both AD and CTE, 9 controls. All were neuropathologically confirmed.	CCL11 levels trended towards significance only in samples with both AD and CTE, compared to controls (p = 0.09).
Cherry et al. 2020 (41)	CCL2	Immunoassay and staining of postmortem DLFC and calcarine cortex	94 RHI-exposed (20 without CTE, 27 low CTE, 47 high CTE); 112 AD (60 low, 28 intermediate, 24 high); 18 non-RHI controls. All neuropathologically confirmed.	CCL2 correlated with increased CTE severity (p < 0.001) and with increased football career length (p < 0.005). CCL2 has a correlation with p-tau, which was independent of Aβ42-status or age.
van Amerongen et al. 2024 (66)	IL-6	CSF analysis from retired football players	104 retired athletes with NBD diagnosis, 76 retired athletes without NBD diagnosis	IL-6 was significantly elevated in subjects with NBD diagnosis. IL-6 levels correlated with various measures of NBD.
Gard et al. 2023 103	IL-2 IL-15 TNF-α TNF-β eotaxin TARC VEGF CXCL10	CSF analysis from living athletes	24 symptomatic athletes with sports related concussions (SRC), 12 healthy controls.	Levels of IL-2, IL-15, TNF-α, TNF-β, eotaxin, and TARC were all elevated in SRC-exposed athletes compared to controls. VEGF levels were significantly elevated in athletes with SRC history. CXCL10 was increased in athletes compared to controls.
Asken et al. 2023 (68)	IL-6 IFN-γ YKL-40	Plasma analysis from living subjects	33 RHI/TES [11 Aβ+, 22 Aβ-], 62 AD (RHI-), 59 healthy controls (RHI-)	RHI/TES had significantly higher IL-6 concentrations compared to controls (Effect size, d = 0.67), and AD participants (d = 0.68). Aβ- RHI/TES had significantly higher IL-6 compared to Aβ+ RHI (d = 1.2), AD (d = 1.1), or controls (d = 1.1).
Di Battista, Rhind, Richards et al. 2016 (42)	CCL2 CCL11 IL-8, Il-12 IL-15, IL-4, IL-10, IL-13	Immunoassay of blood from living athletes	87 college-level athletes including football, field hockey, rugby, basketball, and baseball. Of these, 40 participated in collision sports.	CCL2, CCL11, IL-8, IL-12, IL-15, were quantifiable in majority of participants, ranging from 77 % to 100 %.
Begum et al. 2020 (43)	CCL2 TNFSF14 CX3CL1 IL-7	Plasma analysis of living professional rugby players	18 athletes with single concussion, 5 who were repetitively-concussed, 12 healthy controls	Reduced CCL2 was associated with severity of symptoms (p = 0.043) and increase in number of symptoms (p = 0.013). TNFSF14 levels were reduced in concussed subjects compared to repetitively-concussed subjects. IL-7 levels were higher in repetitively- concussed subjects compared to concussed subjects. CXCL3 was increased less than one week after concussion.
Huibregtse et al. 2020 (54)	CCL11 IL-10	Plasma analysis of 39 living soccer players with heading experience	22 soccer players who underwent a repetitive-heading event, and 17 soccer players who underwent a repetitive-kicking event	No significant increase in CCL11 occurred after repeated-heading event. Increases in CCL11 were significantly correlated with years of heading experience (p = 0.01).
Miner et al. 2024 (74)	IL-6	Plasma analysis of living subjects	180 former football players, 60 asymptomatic unexposed male subjects	IL-6 levels did not significantly differ between RHI-exposed symptomatic subjects and unexposed asymptomatic controls.
Nitta et al. 2019 (67)	IL-6	Serum analysis of football players	857 high school and collegiate football players	IL-6 levels increased within six hours after single concussion. Levels of IL-6 at six hours after concussion were associated with duration of symptoms (p = 0.031).
Alosco et al. 2018 (15)	sTREM2	Immunoassay of CSF from former NFL players	68 former NFL players, 21 non-RHI controls	Levels of sTREM2 were significantly associated with t-tau. sTREM2 strengthened the relation between amount of RHI exposure and t-tau levels
Asken et al. 2022 (84)	NfL GFAP	Ante-mortem plasma analysis from RHI-exposed subjects, with postmortem evaluation	9 RHI-exposed subjects (5 with confirmed CTE).	Plasma levels of GFAP generally showed a longitudinal increase. Baseline levels of GFAP were higher for RHI-exposed subjects compared to healthy controls.
Bernick et al. 2023 (80)	NfL GFAP	Plasma analysis from retired athletes, active athletes, and non-RHI controls	211 active martial arts fighters, 140 active boxers, 69 retired boxers, 52 controls	GFAP levels correlated with cortical and sub-cortical atrophy on MRI, and lower cognitive scores, for retired boxers. GFAP elevation correlated with decreased corpus callosum and thalamic volumes on MRI for active boxers. GFAP levels were highest among retired boxers compared to active boxers, while NfL levels were highest among active boxers compared to MMA fighters.
Shahim et al. 2022 (83)	GFAP	Plasma and CSF analysis of living, symptomatic athletes	28 RHI-exposed professional athletes with persistent postconcussive symptoms, 19 age-matched unexposed controls	Plasma GFAP moderately correlated with CSF GFAP (r = 0.45, p = 0.02)
Bazarian et al. 2024 (81)	GFAP	Plasma analysis of living football players	30 collegiate football players	GFAP increased from pre- to post-game, from 79.69 pg/mL to 91.95 pg/mL (p = 0.008), then to 99.21 pg/mL (p < 0.001) Post-game GFAP changes correlated with reduced functional anisotropy in right fornix (r = -0.59), and adjusted correlations with head impact metrics (r = 0.69–0.74).
Huibregtse et al. 2023 (82)	GFAP	Serum analysis of female water polo players	22 female collegiate water polo players	GFAP increased from week 1 to week 8 of preseason (p = 0.002)

CTE, chronic traumatic encephalopathy; TES, traumatic encephalopathy syndrome; AD, Alzheimer’s disease; RHI, repetitive head impacts; DLFC, dorsolateral frontal cortex; CSF, cerebrospinal fluid; NBD, neurobehavioral dysregulation; SRC, sports-related concussion.

## CCL2

CCL2 is a chemokine involved in regulating monocyte infiltration through the blood-brain barrier (BBB) in response to trauma, and it may be involved in inducing transcriptional alterations in microglia (38). CCR2 deletion or deficiency can also restrict the degree of cognitive dysfunction after TBI (39,40). Despite a general association of CCL2 with tau accumulation as seen in various tauopathies, Cherry et al. reported evidence of CCL2 having a vital role in mediating CTE pathogenesis through microglial activation. By immunoassay, CCL2 in the dorsolateral frontal cortex was found to be elevated in both low and high-stage CTE compared to controls, correlating with severity of CTE pathology. Notably, CCL2 levels correlated with football career length, suggesting a link between CCL2 signaling and RHI exposure duration (41). In fact, the correlation between CCL2 and p-tau, found in both CTE and AD groups, was independent of Aβ42 (Amyloid beta 1-42) levels (41). Thus, it is possible that the tauopathy in CTE is more specifically driven by CCL2. This is supported by the finding that CCL2 was significantly correlated with the density of Iba1- (Ionized calcium-binding adapter molecule 1) positive cells, which itself was found to be associated with the pathognomonic lesion of perivascular tau (41).

Support for CCL2 as a marker of head trauma also comes from other studies. In a blood-based biomarker analysis of university athletes from various contact sports at the beginning of competitive season, female athletes with prior exposure to multiple concussions had elevated serum levels of CCL2 compared to non-exposed controls. In fact, out of 39 markers including IL-1β, IL-6, IFN-γ, IL-10, and TNF-α, CCL2 was the only protein that exhibited a significant relationship with multiple concussion exposure in this group (42). Interestingly, Begum et al. reported reduced CCL2 serum levels to be associated with an increased number and severity of post-concussive symptoms in athletes (43).

However, CCL2 is also strongly associated with AD pathology (44), in addition to multiple sclerosis (47) and stroke (48,49). Consequently, while CCL2 may not have the specificity to CTE that would make it a viable biomarker, its demonstrated involvement in pathogenesis and possible sensitivity to CTE suggests that CCL2 could instead have value as a marker of disease progression. This is supported by the findings of a stepwise increase across RHI exposure and CTE staging continuum, although more evidence is required to validate these results. Because of the role of CCL2 in microglial recruitment and subsequent tauopathy, regulation of this signaling pathway may eventually serve as a therapeutic target in order to minimize the degree of chronic inflammation. However, future investigations should still aim to better characterize the precise involvement of this signaling pathway in the CTE immune signature.

## CCL11

CCL11, or eotaxin-1, is a chemokine that is involved in recruiting eosinophils to sites of inflammation to trigger immune responses (50). Due to its ability to cross the blood-brain barrier, CCL11 can directly contribute to neuroinflammatory mechanisms. In fact, neurons, microglia, and other CNS cell types are known to express receptors for CCL11 (51). In a study that used enzyme-linked immunosorbent assay (ELISA) to assess CSF levels of CCL11 in brain tissue from football players, Cherry et al. found a significantly elevated level of CCL11 in the 23 CTE-confirmed subjects compared to the 50 AD-confirmed subjects and 18 non-athlete control subjects. Of note, the authors determined a significant correlation between CCL11 levels in CTE subjects and football career length. Length of RHI exposure and degree of tauopathy in the dorsolateral frontal cortex were more predictive of CTE diagnosis compared to age (52). However, this study did not include RHI-exposed subjects without CTE; therefore, it is unclear at this point whether CCL11 reflects CTE or RHI exposure independent of disease status. Furthermore, no notable increase in CSF levels of CCL11 among the AD subjects was determined, which corroborates results from other studies (46,53). This would support CCL11 as a promising biomarker that may help differentiate CTE from other pathologies (52).

CCL11 has been observed in the context of sports injury in other studies as well. In the study from Di Battista et al., CCL11 was quantified in serum samples from college-level athletes with and without a history of multiple concussions. Although this chemokine was detected, there were no significant differences in CCL11 levels between those with more than three prior concussions, and those with fewer or none, suggesting that peripheral CCL11 is not persistently elevated in young, asymptomatic athletes despite concussion exposure. Additionally, correlation analyses showed no significant associations between CCL11 levels and time since last concussion, number of prior concussions, or self-reported symptoms.(42). In soccer players, Huibregtse et al. (2020) investigated acute changes in CCL11 following repeated heading events, finding no significant increases in plasma levels after ten head traumas. Despite no group-level acute effects from intervention, exposure duration reportedly contributed to individualized differences in CCL11 within the head trauma group. Although not elevated from baseline, plasma CCL11 levels at 24 hours post-exposure were positively associated with years of heading experience, with an estimated 2.0 pg/mL increase per year of exposure. This suggests a unique sensitivity to cumulative rather than acute RHI exposure (54).

However, there are several challenges with using CCL11 as a biomarker for CTE. While some studies have observed more specific expression in the context of head trauma, other studies have demonstrated elevated levels in AD, Huntington’s disease, and stroke (55). Additionally, CCL11 has also been implicated in normal aging, which complicates its use in older subjects (58). Furthermore, some studies have not observed increases in CCL11 in the context of CTE. In a post-mortem analysis of vitreous humor, samples from CTE subjects did not show significantly elevated levels of CCL11 compared to AD or healthy controls, nor were there differences in CCL11 levels across CTE stages (59). There was only a significant increase of CCL11 in samples from subjects with both AD and CTE pathology when compared to controls, suggesting possible synergistic effects when CTE co-occurs with other neurodegenerative pathologies. Although these results were found in the context of vitreous humor, it will be important to validate CCL11 levels in CSF or plasma in more independent cohorts.

## CCL21

CCL21 or 6CKine, is a chemokine involved in mediating the migration of CCR7-expressing immune cells i.e., T-cells and dendritic cells to lymphoid organs. In the CNS, CCL21 may be upregulated, facilitating lymphocyte migration through the BBB (60). Using an ELISA panel of 71 immune proteins to examine anterior cingulate grey matter samples from patients with various tauopathies, Cherry et al. determined that CCL21 was most strongly correlated with the CTE-confirmed cases, suggesting some specificity to CTE pathogenesis (22). Cherry et al. also examined CCL21 levels between CTE-confirmed and AD-confirmed subjects via post-mortem CSF analysis, showing that there was a significant elevation of CCL21 levels in AD (22). An important caveat of this study was that it only assessed the difference between different types of tauopathies and did not include non-disease control cases. However, the findings suggest CCL21 might be useful to help better segregate related neuropathologies. Elevated CCL21 levels may not only assist with determining suspected CTE, but can also be a point of future investigation in determining how the pathophysiology of CTE might differ from mechanisms seen in other tauopathies. Given its known role in trafficking immune cells across the BBB to propagate inflammation, in addition to its demonstrated specificity to CTE, CCL21 may contribute directly to early immune activation and subsequent CTE pathogenesis. As such, future research should investigate whether CCL21 expression is elevated during early stages of CTE, as this could clarify its role in initiating or sustaining pathology.

There are sparse studies examining CCL21 in the specific context of head trauma, especially in humans. In fact, the existing neurotrauma data on CCL21 primarily stems from animal model studies on spinal cord injury (SCI) (61,62). Of note, one human-based study on SCI exists to date, reporting a negative correlation between serum levels of CCL21 and neuropsychological test scores in SCI patients, suggesting CCL21 as a possible correlate for post-injury cognitive dysfunction (63). Consequently, this gap in the literature highlights the need for future studies assessing bio-fluid levels of CCL21 in relation to head trauma, particularly in living subjects with RHI exposure and during post-mortem evaluation.

## IL-6

IL-6 is a multifunctional cytokine critically involved in the neuroinflammatory cascade. In the CNS, IL-6 is produced primarily by activated microglia and astrocytes following trauma, where it promotes leukocyte migration and cytokine signaling (64,65). A recent investigation used a CSF immunoassay on retired football players for inflammatory proteins including IL-1β, IL-6, IL-8, IL-10, TNF-α, and C-reactive protein. Of these markers, only IL-6 was significantly elevated in subjects who had neurobehavioral dysregulation (NBD), compared to retired players without an NBD diagnosis (66). This is noteworthy since NBD, referring to behavioral changes and dysregulation of emotion, is a prominent clinical finding in the proposed criteria for TES (traumatic encephalopathy syndrome). Van Amerongen et al. found IL-6 to be correlated with the overall NBD score, as well as with subdomains including impulsivity, emotional dysregulation, and affective lability. Interestingly, IL-6 appeared to have a selective relationship with certain behavioral traits, as it did not correlate with the explosiveness domain of NBD or with measures of cognitive performance. None of the tested markers in the CSF, including IL-6, were associated with RHI proxies i.e., football career length (66). This points to IL-6 as a potential sensitive marker, particularly compared to other inflammatory cytokines, for monitoring progression of NBD symptoms in those persons at risk for CTE.

Similarly, serum levels of IL-6 were found to correlate with clinical symptom severity after a sports-related concussion, as it remained elevated at 14 days post-injury (67). Additionally, IL-6 levels in the plasma were significantly elevated in RHI/TES patients compared to healthy controls and AD patients without RHI exposure. Furthermore, RHI/TES patients who were negative for Aβ-PET negative, i.e. negative for Aβ in positron-emission tomography, still had significantly higher IL-6 levels than RHI/TES patients with Aβ+ status and all other groups, suggesting that IL-6 elevation is more reflective of chronic RHI-induced inflammation than of any potential co-morbid Alzheimer’s pathology (68). While TES criteria have not been pathologically validated, these findings suggest that IL-6 might play a role in neuropsychiatric symptoms observed in patients with RHI exposure, including those who may eventually develop CTE. However, IL-6 plasma levels have also been previously linked to disinhibition in frontotemporal dementia (FTD) (69), apathy in AD (70,71), and manic symptoms in bipolar disorder (72). Thus, IL-6 may non-specifically contribute to NBD symptoms in various neurologic or psychiatric conditions, including CTE. Future investigations could benefit from directly assessing IL-6 as a possible pathological correlate for NBD in CTE, such as by comparing postmortem findings with retrospective review of clinical symptoms.

However, not all studies have found success with IL-6 as an RHI-related biomarker. Parkin et al conducted an immunoassay of blood-based immune markers up to 3 months after pediatric concussion, observing that patients with normal recovery exhibited the same gradual decrease in IL-6 expression as patients with persisting symptoms showing concentration difficulty as well as cognitive and behavioral deficits (73). That said, the symptom categories in this study were broadly defined and likely included general post-concussive complaints like dizziness and headache, rather than specific components of NBD. Non-specific symptom classification may have obscured potential relationships between IL-6 and behavioral phenotypes when considering the findings from Van Amerongen et al. that suggest IL-6 to have a selective association with NBD subdomains (74).

Additionally, IL-6 plasma levels in former football athletes did not significantly differ between RHI-exposed symptomatic subjects and unexposed asymptomatic controls. Furthermore, IL-6 plasma levels were not significantly associated with RHI proxies including years of play and age of first exposure (74). To address uncertainty regarding IL-6 as a viable marker for symptomology, future examinations should longitudinally assess IL-6 in RHI-exposed subjects alongside clinical phenotypes, aiming to determine if IL-6 levels reliably track symptom onset, persist independently of clinical progression, or reflect non-specific post-traumatic inflammation.

## GFAP

Considering the role of astrocytes in facilitating neuroinflammation, some studies have assessed GFAP in relation to RHI. GFAP is a filament protein in the cytoskeleton of astrocytes and provides structural support (75). Since GFAP is upregulated during astrogliosis, elevated GFAP levels may indicate a neuroinflammatory response, or an injury to astrocytes (76). GFAP is also a potential predictor of concussion status or increased RHI severity, albeit more-so in an acute post-TBI timeframe (77,78).

Although postmortem studies provide evidence of altered GFAP expression in CTE (16,79), fluid biomarker studies in pathologically confirmed CTE are rather limited. However, GFAP elevations have been observed in RHI-exposed populations who may be susceptible to CTE. In a longitudinal cohort study of professional boxers and mixed martial art fighters, Bernick et al. (2023) measured plasma levels of GFAP annually over one to four years of follow-up, examining the association of GFAP levels with structural MRI data and cognitive outcomes. Longitudinal increases in GFAP levels were significantly associated with decreased volume of certain brain regions on MRI, including hippocampus and thalamus, in addition to enlarged lateral ventricles. These structural changes also correlated with declines in processing speed, memory, and reaction time, suggesting that GFAP is a viable marker for long-term neurodegenerative progression in RHI-exposed populations (80).

Additional studies further support the relevance of GFAP as a marker of astroglial activation following RHI. In a study from Bazarian et al., serum GFAP was measured in collegiate football players before, immediately after, and 45 minutes post-game. Although no athletes sustained a diagnosed concussion, GFAP levels increased significantly after the game, with magnitudes of increase correlating with both helmet-recorded head impact exposure and reduced white matter integrity measured via diffusion tensor imaging. These associations remained significant even after adjusting for physical exertion, suggesting that acute GFAP elevations may reflect subclinical astrocytic injury related to repetitive, non-concussive impacts sustained during a single game (81). Furthermore, in a study on collegiate women’s water polo players, serum GFAP concentrations were assessed over an eight-week preseason period. The authors reported a significant linear increase in GFAP over the study period, while no acute changes in GFAP were observed following scrimmages, suggesting that astroglial activation may accumulate over time even in the absence of concussion diagnoses. However, this longitudinal GFAP increase was not significantly associated with cumulative head impact burden calculated based on acceleration metrics from instrumented mouth guards (82). These findings support the notion that while GFAP can increase longitudinally in RHI-exposed populations, the utility of GFAP as a specific marker of head impact burden, particularly in subconcussive settings, may be limited.

Shahim et al. evaluated CSF and plasma levels of GFAP in a cohort of RHI-exposed professional athletes with persistent symptoms. Plasma GFAP levels showed a moderate correlation with CSF GFAP levels, but plasma levels did not significantly correlate to symptom severity or number of prior concussions. In addition, serum GFAP levels did not significantly differ between RHI-exposed subjects and unexposed controls, and serum GFAP levels did not relate to BBB integrity measured via the CSF/serum albumin ratio. Thus, GFAP may have limited utility in the later stages of post-RHI neurodegeneration long after initial exposure, suggesting a temporal window where astrocytic markers could be more sensitive to injury (83). As such, future studies may aim to better characterize the long-term trajectory of serum GFAP from RHI exposure to post-mortem evaluation.

Furthermore, Asken et al. (2022) examined plasma GFAP levels in a clinicopathological cohort of nine RHI-exposed individuals who were followed to autopsy. Three out of five subjects with longitudinal data had a steady increase in GFAP concentration over time, ranging from two to seven years, in addition to having higher levels at baseline compared to healthy controls. Among the five cases with autopsy-confirmed CTE, elevated GFAP levels tended to co-occur with imaging evidence of medial temporal atrophy and cognitive decline. CTE was the primary neuropathological diagnosis in two cases in which persistently high GFAP levels were observed. While these findings suggest a possible association between GFAP elevation and CTE pathology, the presence of frequent co-pathologies such as AD or TDP-43 proteinopathy, underscores that GFAP is not a specific marker of CTE and may instead be implemented as a sensitive marker for astrocytic activation in the context of neurodegeneration (84). Taken together, these findings underscore that while GFAP may be a sensitive indicator of acute astroglial responses to RHI, including subconcussive impacts, it could be subject to temporal and individual variability. Although promising, the utility of GFAP as a long-term biomarker of neurodegenerative risk will likely require longitudinal tracking and integration with symptom trajectories and other fluid markers to improve specificity for CTE.

## Additional prospective markers of CTE

The above mentioned neuroinflammatory candidate markers have been examined to a limited extent for their application to CTE pathology. In addition, there are several other immune mediators that may be considered for future post-mortem investigations. In general, these immune proteins have been primarily assessed through mouse models, as well as in RHI-exposed living subjects for some cases. Yet, there is limited understanding of how exactly they fit into the signature for CTE.

Future investigations could assess the specificity of these markers to CTE by conducting comprehensive panels of immune proteins in post-mortem tissue. It would be particularly beneficial to directly compare CSF or serum levels of these potential markers between CTE and other neurodegenerative diseases that may result from head trauma i.e. AD. The prospective markers are discussed below, with pertinent findings from human-based studies also included in **Table 1**. Because of the limited literature of these markers, we propose novel proteins that could be worth examining, as the latter have not yet been applied to RHI at the human level or to post-mortem CTE.

## sTREM2

Based on the vital role of microgliosis in neuroinflammation, microglial markers may have some viability as diagnostic or therapeutic targets for CTE, such as soluble triggering receptor expressed on myeloid cells 2 (sTREM2). This receptor, upregulated in activated microglia, has been implicated in various neurodegenerative diseases due to its role in regulating microglial survival, proliferation, and phagocytosis (85). It is worth noting that triggering receptor expressed on myeloid cells (TREM2) refers to a transmembrane protein found in microglia which is involved in a variety of functions, such as facilitating microgliosis and regulating lipid metabolism (86). Proteolysis leads to release of sTREM2 in CSF. The protein sTREM2 is generally shown to be associated with tauopathy and has been primarily studied in the context of AD, but its overall function is less understood (87).

By analyzing CSF samples from RHI-exposed retired NFL players, Alosco et al. determined that levels of sTREM2 significantly correlated with the amount of total tau (t-tau) in the RHI-exposed group, despite the absence of group-level differences in sTREM2 levels between the athletes and control subjects (15). Despite an insignificant association between sTREM2 and cumulative RHI exposure, regression analysis showed that sTREM2 levels increased the strength of association between RHI exposure and t-tau levels (15). This suggests some early involvement of sTREM2 in facilitating CTE pathology in response to RHI. A potential limitation to implementing sTREM2 as a marker for CTE is the extensive prior evidence of sTREM2 having a strong association with AD pathology (88). Consequently, future investigations may focus on assessing sensitivity and specificity of sTREM2 or other potential microglial markers in CTE in comparison to cases of AD or other tauopathies.

## Pro-inflammatory mediators

In addition to IL-6, other pro-inflammatory cytokines related to head impact exposure may serve as potential markers of CTE progression. Gard et al. found that among athletes with prior exposure to sports-related concussions, CSF analysis revealed a significant increase in IL-2, IL-15, TNF-α, TNF-β, eotaxin, and TARC levels compared to those in control athletes. It is possible that these protein levels increase with post-concussive symptoms, suggesting a possible role of these cytokines in early phases of injury-related pathology (104). However, considering that the study included healthy controls without AD for comparison, it remains unclear whether these elevations represent acute or persistent changes. Notably, other studies have reported more limited associations between cytokines level and RHI exposure. In one blood-sample analysis of college-level athletes, there was no significant correlation between participation in collision sports and levels of IL-8, IL-12, IL-15, or TNF-α. In fact, only peripheral levels of tau were significantly associated with participation in collision sports, suggesting that the various cytokines may not show consistent peripheral elevation in response to trauma (42). In addition, van Amerongen et al found no significant association between CSF levels of TNF-α and IL-1β and NBD scores in retired football players, suggesting that these markers may not be related to clinical symptom expression (66). Plasma levels of IFN-γ were also not found to be significantly increased in living subjects with RHI/TES, when compared to AD and controls without RHI exposure (68). While not directly applicable to CTE due to the lack of validation of TES to pathology, these findings suggest that cytokine expression following head trauma may be context-dependent, varying by injury time course, sampling method, and clinical phenotype.

Begum et al. conducted a serum-based comparison of inflammatory markers between athletes after single concussion and athletes who were repetitively concussed. The authors found that serum levels of TNFSF14, belonging to the TNF superfamily, and IL-7 were significantly higher in repetitively concussed players than in players after single concussion, suggesting that the two proteins may reflect inflammatory responses to repeated injury (43). These findings offer preliminary support to use TNFSF14 and IL-7 in order to distinguish immune profiles between single TBI and RHI. While the study defined “repetitively concussed” as two concussions within the span of three months, which may not fully represent the accumulation of minor trauma seen in RHI, the observed differences still point toward an immunological distinction between acute and cumulative injury states. As such, future studies may benefit from determining how TNFS14 and IL-7 might correlate with proxies of RHI.

Other pro-inflammatory aspects of the CTE immune signature that have not yet been explored in CTE include DAMPs (damage-associated molecular pattern) such as high mobility group box protein 1 (HMGB1). HMGB1 is a DNA binding protein that can be actively secreted by leukocytes in response to cytokine activation, or it may be passively released via neuronal death (91,92). In addition to being a marker of neurodegeneration, HMGB1 can act as a DAMP and mediate further inflammation (93). While HMGB1 overexpression is also common to conditions like epilepsy or AD, an analysis of various HMGB1 isoforms and their potential specificity for different neurodegenerative states could help clarify the immune signature for CTE (94,95). Likewise, heat shock proteins such as HSP70 or HSP90 are other proteins that are released during cell death and may trigger more inflammation by functioning as DAMPs, thus possibly serving as a measure of cellular stress in response to post-RHI neuroinflammation (96,97). Although HMGB1 and heat shocks proteins have yet to be studied in either RHI or CTE, future investigations analyzing their precise role in the CTE immune signature, such as through immunoassay or serum analysis, may help to clarify their usefulness as markers.

Furthermore, YKL-40 may be a reliable measure of astrocytic-mediated inflammation in suspected CTE, since it has been shown to have upregulated expression by astrocytes and microglia during trauma, including in the chronic neuroinflammatory state (98). While Asken et al. 2023 did not find a significant elevation of YKL-40 in living subjects with RHI exposure, it could be worth assessing this finding in the post-mortem setting (68). Notably, CSF levels YKL-40 have also been shown to be elevated in autopsy-confirmed AD, including some correlation with patterns of Aβ deposition (99). Thus, it may be relevant to conduct a comparison between CTE and AD tissue, seeking to determine whether YKL-40 can help discriminate between those two pathologies.

## Anti-inflammatory mediators

The role of anti-inflammatory proteins in post-RHI immune processes is even less understood than that of pro-inflammatory cytokines. However, since anti-inflammatory proteins tend to resolve inflammation and to promote tissue repair, related investigations may help to elucidate some of the nuances in CTE progression, including any immune-related mechanisms in response to secondary injury from inflammation. While several human studies have examined anti-inflammatory factors like IL-10, IL-4, and IL-13, few correlations could be identified (54) (42). However, there have been reports in animal studies that TGF-β could be elevated six months after repetitive head trauma (100). Overall, while there is less support for anti-inflammatory proteins as novel biomarkers for repetitive head trauma, more work is needed to better clarify targets.

## Discussion and future directions

Emerging evidence points to immune mediators as more specific CTE biomarkers. Given that inflammation contributes to CTE pathology, immune mediators that could be unique to CTE’s inflammatory signature, such as CCL11 and CCL21, are worth further exploration (22,52). However, a definitive answer on the reliability of immune mediators depends on a few challenges and limitations that will first need to be addressed in future studies. One of the challenges is the need to account for the prolonged latent period between RHI exposure and CTE symptom onset. The progressive nature of chronic inflammation in CTE complicates the identification of immune markers that are involved over the course of disease development. Additionally, promising markers like CCL11 and CCL21 have been primarily assessed post-mortem, making it difficult to translate these findings to living patients.

An overarching challenge with developing ante-mortem biomarkers from immune mediators is that, currently, CTE can only be definitively diagnosed post-mortem, with the 2021 consensus criteria subject to future refinement (101). As such, many of the studies on CTE are essentially just a snapshot in time and are limited in addressing full “causation” of the target factor versus apparent correlation. To address this, future studies should track immune markers in living RHI-exposed individuals over time, followed by CTE status confirmation via autopsy. This approach is essential not only for offering clearer insights into the temporal dynamics of the CTE inflammatory profile but also for revealing additional novel biomarkers that distinguish CTE from other diseases. Prospective studies could measure general injury markers such as NfL or GFAP in RHI-exposed living individuals to find subjects in the early stages of neurodegeneration who are not yet symptomatic. Subsequently, there can be an assessment of immune profiles via CSF- or serum-based analysis during life, followed by pathological confirmation. This would allow researchers to assess whether fluid-based detection of immune proteins corresponds to confirmed CTE pathology, helping bridge fluid markers to tissue-based diagnostic criteria. The overlap of some immune mediators between CTE and other neurologic conditions such as aging or AD further complicates the potential of immune mediators as biomarkers for CTE. For instance, cytokines like CCL11 and IL-6 can be elevated in normal aging and in psychiatric disorders, respectively, limiting specificity to CTE (58,69). However, despite lack of specificity for some cytokines to CTE, implementing this approach may nevertheless allow researchers to identify changes in immune mediators across the disease course, including prior to symptom onset and early and late stage CTE.

Inflammatory markers should be viewed as central though not sole components of a comprehensive CTE biomarker profile. While they may not provide absolute specificity on their own, immune mediators may enhance the disease-stage resolution of diagnostic panels when integrated with other biomarkers such as tau isoforms or general injury markers. For example, while t-tau may not distinguish CTE from other tauopathies, specific tau epitopes like p-tau202 and p-tau231 may be unique to CTE and warrant further study in both living patients and in post-mortem comparisons with other neurodegenerative diseases (18,102). Overall, continued validation of immune markers like CCL21 and their potential role in CTE is needed through fluid-based analyses in both post-mortem and ante-mortem settings.

**Figure 1:**
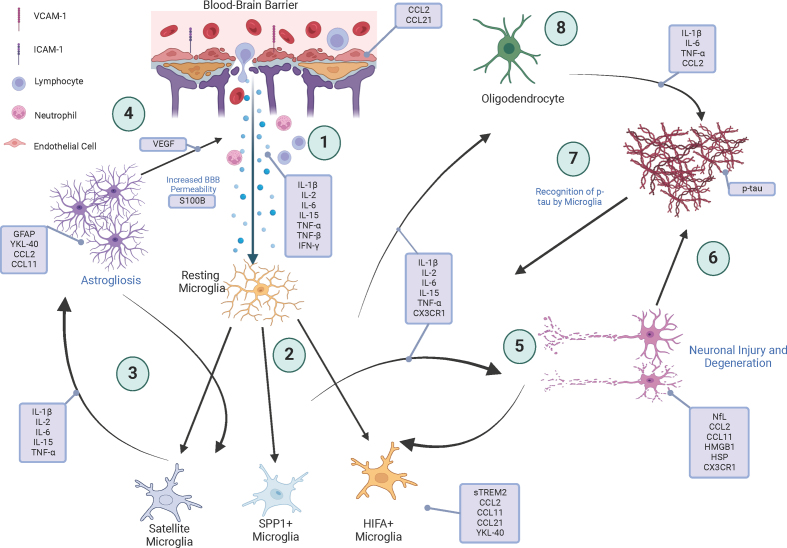
Inflammatory pathways contributing to the immune signature for CTE are highlighted, including key cellular interactions and sources of potential biomarkers. The schematic depicts the sequential activation of endothelial cells, microglia, astrocytes, neurons, and oligodendrocytes, through which chronic inflammation and neurodegeneration is maintained. Created with BioRender.

In **Figure 1**, we provide a schematic overview of the inflammatory pathways comprising the immune signature where prospective CTE biomarkers might fit. 1) Repeated head trauma damages vasculature, leading to increased BBB permeability and infiltration of lymphocytes and pro-inflammatory cytokines like IL-1β and IFN-γ. At this point, CCL2 and CCL21 may be upregulated by endothelial cells. 2) Microglia are activated by cytokines, proliferating into phenotypes including satellite, SPP1+, and HIFA+ microglia. Microglia then secrete factors such as CX3CR1, TNF-α, and IL-6 that propagate inflammation through stimulation of other neural cell types. Microgliosis may correlate with candidate markers CCL2, CCL11, CCL21, and YKL-40. 3) Astrocytes proliferate and release inflammatory cytokines e.g. IL-1β, IL-2, IL-6, and IL-15) that further activate microglia. 4) Reactive astrocytes contribute to an increased BBB permeability, exacerbating inflammation. YKL-40, CCL2, and CCL11 may be released by activated astrocytes. 5) The neuron-microglial crosstalk occurs, where microglia damage neurons through cytokine release, and neurons in turn secrete mediators including ROS that promote more microgliosis. Markers associated with this event could include CCL2, CCL11, HMGB1, HSP, and CX3CR1. 6) Next, p-tau is released from damaged neurons and accumulates. 7) Tau deposition is then sensed by microglia which are further activated. 8) When damaged, oligodendrocytes might cause neuronal injury through secretion of cytokines such as IL-1β, IL-6, TNF-α, CCL2 and also axonal degeneration. While several of these processes might also be involved in other diseases or even in single TBI, the cellular arrangement, brain region affected, regional spread, timing, and age of onset can be distinct in the context of CTE. These are important processes to consider when utilizing biomarkers to help discriminate and identify pathology. For example, although CCL2 might also be implicated in AD, elevated CCL2 levels found in individuals in their 30s may rather point towards CTE, given that AD changes typically start around the age of 60.

## Conclusion

As it is the case in many neurodegenerative diseases, neuroinflammation has a critical role in CTE pathogenesis and disease trajectory. As such, neuroinflammatory changes might be useful to foster biomarker development. In this review, we have highlighted several prospective fluidic neuroimmune biomarkers that might be useful to identify CTE during life. CCL21, CCL11, CCL2, IL-6, and GFAP have all shown some promise but have yet to be fully validated. However, several limitations surrounding specificity arise as these factors are also involved in other neuropathologies. Therefore, it is not likely that a single biomarker will be sufficient for accurate CTE detection. In genetics, examining multiple genes together to identify “gene signatures” has shown to be a better method to identify complex changes and disease-specific effects. In that same line of ideas, it is likely that biomarker panels consisting of several neuroimmune proteins will be able to specifically identify diseases more efficiently as previously suggested (103). In addition, it will be useful to also include other variables such as clinical symptoms, demographic details, athletic history, and imaging results to further help increase the specificity of CTE detection. These types of clinical-pathologic correlation studies are currently ongoing and hoped to bridge clinical details with neuropathologically confirmed disease status and biomarker data.

In conclusion, the work presented here highlights that our continued understanding of neuroinflammation offers the exiting ability to improve existing detecting techniques and to increase our future ability to identify CTE during life.

## Authors' contributions

GSB: conceptualization, writing (original draft), writing (review and editing). SA: writing (original draft). JS: writing (original draft). FL: conceptualization. AEB: writing (review and editing). KWT: writing (review and editing). JDC: conceptualization, writing (original draft), writing (review and editing). All authors have read and approved the final version of the manuscript.

## Conflict of interest statement

The author declares no conflict of interest.
